# *Trypanosoma cruzi* transmission in the wild and its most important reservoir hosts in Brazil

**DOI:** 10.1186/s13071-018-3067-2

**Published:** 2018-09-06

**Authors:** Ana Maria Jansen, Samanta Cristina das Chagas Xavier, André Luiz Rodrigues Roque

**Affiliations:** Laboratório de Biologia de Tripanosomatídeos, Instituto Oswaldo Cruz/FIOCRUZ, Av. Brasil 4365, Rio de Janeiro, RJ CEP. 21040-360 Brazil

**Keywords:** *Trypanosoma cruzi*, Wildlife reservoirs, Opossums, Primates, Carnivores, Transmission cycle

## Abstract

*Trypanosoma cruzi* (Kinetoplastea: Trypanosomatidae) infects all tissues of its hosts, which along with humans, include hundreds of mammalian species in the Americas. The epidemiology of *T. cruzi* has been changing in that currently the majority of the cases and/or outbreaks of Chagas disease occur by the ingestion of comestibles contaminated by *T. cruzi* metacyclic forms. These cases/outbreaks occur in distinct regional scenarios, mainly in the Amazon biome and are related to the local interaction mode of humans with their surroundings, as well as with the overall local ecological peculiarities. As trypanosomiasis caused by *T. cruzi* is primarily a zoonosis, understanding the variables that influences its transmission in the wild as well as the role played by the extant fauna in the maintenance of the parasite, is critical in establishing control measures. Here, we present the results of our studies of *T. cruzi* infection of free ranging wild mammalian fauna in the five biomes of Brazil, a country of continental dimensions. From 1992 up to 2017, we examined a total of 6587 free-ranging non-volant wild mammal specimens. Our studies found that 17% of mammals were seropositive and 8% of all animals displayed positive hemocultures indicative of high parasitemia and, consequently, of infectivity potential. We observed that opossums, mainly *Philander* spp. and *Didelphis* spp., the coati *Nasua nasua*, the capuchin monkey *Sapajus libidinosus* and the golden lion tamarin *Leontopithecus rosalia*, were mammal taxa that demonstrated higher rates of positive hemocultures. Additionally, *Didelphis* spp. demonstrated to be a competent bioaccumulator of TcI diversity. Chiroptera were distinguished for hosting the greatest diversity of species and genotypes of *Trypanosoma* spp. Additionally the observation of the higher host range of some *Trypanosoma* spp., shows the need to reassess the ecology of representatives of the taxon. Altogether, our results showed that each locality, may display distinct enzootiological and epidemiological scenarios that must be taken into account when it comes to establishing control and/or clarification campaigns of the local population.

## Background

The current rapid anthropogenic environmental changes, in addition to the uncontrolled human and animal migrations within an increasingly globalized world, act powerfully as parasite dispersers. This is the case of trypanosomiasis caused by *T. cruzi* that in humans can result in Chagas disease, currently considered a global health challenge [1].

*Trypanosoma cruzi* is a true generalist and highly successful parasite in that it is able to infect almost all cell types of hundreds of mammal species distributed from southern USA to the south of Argentina. This parasite species is transmitted by dozens of exclusively hematophagous triatomine bug species (insects of the order Hemiptera), in which the parasite establishes permanent infections. In its mammalian hosts, *T. cruzi* also establishes stable and long-lasting infections, that display peculiarities inherent to the different host species and even individuals and other multiple factors, such as the parasite subpopulations, infection route, host nutritional status and concomitant infection with distinct *T. cruzi* DTUs (discrete typing units) or other parasite taxa [2].

Trypanosomiasis caused by *T. cruzi* is primarily an ancient enzootic parasitosis of wild mammals and its most probable main dispersion strategy in nature is the oral route, by predation on both infected vectors and mammals. Consequently, *T. cruzi* transmission in the wild is deeply immersed in trophic nets [2, 3]. Additionally, the classical contaminative route by metacyclic forms eliminated in infected triatomine feces may frequently occur in animals in their shelters and nests. It is also probable that the contaminative transmission by metacyclic forms of the scent gland of didelphid marsupials [4] represents an efficient transmission mechanism in the wild, but this has not been evaluated so far. This transmission route could have been important in the wild before hemipterans developed hematophagic habits [5]. The competence to infect all mammalian nucleated cells in any tissue as well as the diverse infectious forms, also represent a powerful mechanism of the maintenance of *T. cruzi* in nature. In addition to these evolutionary advantages, *T. cruzi* also displays a huge biological plasticity, probably as the result of its impressive biological, biochemical and molecular heterogeneity. Understanding the origin of *T. cruzi* heterogeneity still represents a challenge: although the population structure of *T. cruzi* is mainly clonal, mitochondrial introgression and hybridization events occur intra and inter-*T. cruzi* DTUs [6]. The frequency with which these phenomena occur in nature and their importance as promoters of *T. cruzi* diversity has been a highly discussed issue for decades. Currently, the set of these *T. cruzi* subpopulations were subdivided into seven taxonomic units or DTUs, namely TcI-TcVI and Tcbat. The latter, formerly assumed as restricted to bats, has been described infecting pre-Columbian human mummies [7, 8]. The attempts that have been made to associate a given subpopulation of the parasite with the distinct forms of human disease, the ecology of its wildlife host species, the biome of its occurrence and vector species have not yet resulted in conclusive associations. If any association exists between a given parasite subpopulation and specific symptoms of the disease or reservoir, only long-term studies of a representative number of *T. cruzi* isolates of representative mammalian species of the different biomes and habitats will establish this. Currently, however, studies have been conducted with samples that are obtained usually on only a single occasion from a single infected individual. These samples are then subjected to selective pressures inherent to isolation and maintenance methods. In addition, representative *T. cruzi* isolates of representative mammalian species present in all habitats where the parasite occurs are lacking. Altogether, we are sure that these gaps result in huge biases.

## After all, what constitutes a reservoir?

Defining what constitutes a reservoir is a practical and theoretical challenge, and there are currently many definitions [9, 10]. Our concept of a reservoir is based on that proposed by Ashford [11], according to which a reservoir, or rather a reservoir system, is the species or the assemblage of species that support the maintenance of a parasite in nature in a sustainable way in a given space scale. Actually, among the several definitions, this seems the best-fit for a complex taxon as in the case of *T. cruzi*. Indeed, this multi-host parasite establishes peculiar and dynamic interaction patterns with each of its hundreds of host species, which therefore play different roles in the parasite transmission network of the different habitats and biomes of its occurrence.

The host’s capacity of being infective to its vectors, under natural conditions, depends on several factors, the most important being the concentration of parasites in the mammalian host’s blood (i.e. the parasitemia). That is, a mammalian host displays its maximum infectivity potential during the period in which it maintains a sufficient amount of circulating parasites to be ingested during the blood meal of the vector, ensuring its dispersion and maintenance in nature. The periods of high parasitemia vary among host species and individuals. The currently known variables that determine the duration of parasitemia in a given animal species (or even individual) include at least parasite genotype, nutritional status, infection route and concomitant infection. A combinatorial analysis of all the variables might result in an infinite number of possibilities. The competence of a mammalian host in infecting vectors was elegantly termed by Brunner et al. [12] as “realized reservoir competence”.

Among the points that should be taken into account when evaluating a given mammalian species as a reservoir, is its precise taxonomic classification. In fact, even cryptic species may present significant differences of the course of the infection with the same species of parasite as is the case of *Thrichomys laurentius*, a caviomorph rodent species of the semi-arid caatinga in northeast Brazil, and *T. fosteri*, a species from the Pantanal, a flood plain region in west-central Brazil. Experimental infections with *T. cruzi* in these two rodent species showed that *T. fosteri* demonstrated a significantly higher and long-lasting parasitemia and much more severe histopathological lesions [13]. These rodent species, which are morphologically absolutely indistinguishable, demonstrated to manage *T. cruzi* infection in totally different ways and consequently play a very different role in the maintenance of *T. cruzi* in the natural environment, as was later demonstrated [14, 15].

Evaluating the infectivity potential of a mammal species is a challenging task since it depends on a long-term follow-up of naturally or experimentally infected animals. In the latter case, the great challenge is the difficulty in rearing and maintaining wild mammal species in animal facilities. Experimental infections in conventional laboratory animal models are not representative and do not mimic infection conditions in the wild. In order to fully understand a reservoir system, it is important within the area of study to take an inventory of the local species, to analyse their population structure and to assess the taxonomic and beta diversity of an area.

One of the major obstacles to the study of wild reservoirs is the lack of species-specific reagents for serological tests. These tests are indispensable because they detect infected animals which have low, undetectable parasitemia, as shown by negative parasitological tests, i.e. low infectivity potential. Ideally two serological tests and two parasitological tests with distinct sensitivity should be employed.

In this review article, we summarize the data concerning wild reservoirs of *T. cruzi* collected during the last 20 years by our research group. We used data exclusively collected by our group as a way of making these data comparable. Moreover, we used the same techniques that we employed after establishing standard operating procedures (SOP) in our laboratory (Labtrip). The technical team, if renewed, received proper training by our staff before assuming any procedure as a way to maintain quality and repeatability. Thus, the methods employed in all steps were the same and are, therefore, comparable. In short: (i) blood cultures were performed in two tubes with 300 μl of blood in each that were examined fortnightly for up to five months; (ii) serology was performed through indirect immunofluorescent assay tests (IFAT), according to Camargo [16] Epimastigotes of *T. cruzi*, strains F (DTU TcI) and Y (DTU TcII) were grown in NNN/LIT medium to exponential phase and subsequently washed. Whole epimastigotes of each strain were mixed in equal proportions and employed as antigen in the IFAT assays. The reaction was revealed by species or specific family conjugate]; and (iii) molecular characterization at the DTU-level by means of two algorithms, mini-exon with RFLP and, more recently, nested-PCR for the *18S* gene followed by sequence analysis. In this sense, all bat samples, as well as original isolates from other mammals not previously characterized in DTUs level, were recharacterized by the nested PCR protocol [17]. The complete methodology, as well as some of the results that were previously published, are described elsewhere [14, 15, 18–30].

Longitudinal studies are labor-intensive, logistically challenging and very expensive; however, they are the most informative. For example, in Brazil, the greatest infectivity potential of *T. cruzi* was observed in free-ranging wild Didelphimorphia (mainly *Didelphis* spp.), Primates and Carnivora. These observations were the result of 20 years of field studies in the different Brazilian biomes [14, 15, 18–30].

The infectivity potential, however, is not taxon-inherent and a fixed trait, as already mentioned above. A four-year study of the sylvatic transmission cycles of *T. cruzi*, performed in Argentina, concluded that cingulate and marsupials other than the genus *Didelphis* spp*.* displayed higher infectivity potential in comparison to *Didelphis albiventris*, classically described as the main *T. cruzi* reservoir [31]. Concerning domestic animals, dogs in Santiago del Estero (Argentina) demonstrated to be highly infective [32] in contrast to dogs in Brazil that rarely display high parasitemia [33, 34].

## The *T. cruzi* enzootic transmission cycle and its idiosyncrasies

*Trypanosoma cruzi* enzootic transmission has been demonstrated to occur all over Brazil. Table 1 shows that 17% of the 6431 examined wild mammals had been exposed to *T. cruzi* infection as revealed by serological methods. Concerning Chiroptera, given their particularities, besides being the ancestral hosts and harboring numerous species of the *T. cruzi* clade, data referring this taxon are being presented in a separate table.

**Table 1:** *Trypanosoma cruzi* natural infection in free-living wild mammals of Brazilian biomes: consolidated results showing the total number of non-volant mammalian orders, genera and species examined by blood cultures and serological tests (indirect immunofluorescence test, IFAT)

Biomes	No. of orders	No. of genera	No. of species	HC(+)/ specimens tested (%) [95% CI]	IFAT(+)/ specimens tested (%) [95% CI]	*χ*^2^-value^a^	*P*-value
Amazon Forest	7	33	31	90/402 (22) [18–26]	168/398 (42) [37–47]	35.968	<0.0001
Atlantic Forest	5	44	53	205/2416 (8) [7–9]	340/2375 (14) [12–15]	40.388	<0.0001
Caatinga	5	24	23	99/1005 (10) [8–11]	225/952 (24) [20–26]	67.235	<0.0001
Cerrado	7	54	67	47/1973 (2) [1, 2]	191/1838 (10) [10–12]	132.321	<0.0001
Pampa	4	10	8	0	15/106 (14)	16.142	
Pantanal	5	27	19	75/791 (9) [7–11]	155/762 (20) [17–23]	36.278	<0.0001
Total				516/6587 (8)	1094/6431 (17)	762.23	<0.0001

^a^*df* =1

Difference between the rates of animals with positive blood cultures and positive serology among biomes were tested by ANOVA (*F* = 12.61, *P* = 0.0075). Comparison between the rates of animals with positive blood cultures and positive serology among biomes using Chi-square test (HC: *χ*^2^ = 34.532, *df* = 5, *P* < 0.000; IFAT: *χ*^2^ = 30.36, *df* = 5, *P* < 0.0001).

*Abbreviations*: CI, confidence interval; HC(+), number of specimens with positive hemocultures; IFAT(+), number of specimens with positive IFAT test

Positive hemocultures, i.e. infectivity potential, was observed in 8% of all 6587 examined animals distributed in all biomes. These data suggest that only 8% of individuals with infective competence are sufficient to maintain the widely dispersed and complex cycle of *T. cruzi* transmission. The lack of species-specific reagents impaired the performance of serological tests of all mammalian taxa. Even so, the sample of individuals that we were able to test was expressive and showed that 17% of them were seroreagent for *T. cruzi* showing that the infection of the majority of the infected mammals is expressed by positive serology (*χ*^2^ = 197.36, *df* = 1, *P* < 0.0001). It is worth noting that infected animals were distributed in all biomes. The rate (percentage) of mammals displaying positive blood cultures was considerably higher in the Amazon in comparison to the other biomes (Table 1).

All mammalian orders (Artiodactyla, Chiroptera, Primates, Carnivora, Rodentia, Cingulata, Pilosa and Didelphimorphia) demonstrated to be involved in the transmission cycle of *T. cruzi* to a greater or lesser degree and in distinct time- and space scales (Tables 2, 3, 4, 5, 6 and 7). Thus, the lowest number of infected mammals has been observed in the Cerrado, contrasting with the Amazon biome, where *T. cruzi* infection rates were the highest. The following taxa may be considered as key reservoir taxa due to the higher rates of parasitemia (as expressed by positive hemocultures) and competence in maintaining a higher diversity of *T. cruzi* DTUs, as well as other *Trypanosoma* species: Primates, Didelphimorphia, Chiroptera, and the Carnivora species *Nasua nasua.* Mixed *T. cruzi* DTU infections are frequent, notably in generalist (concerning habitats and feeding habits) mammal species [2]. That means that, besides environmental conditions (habitats, climate, anthropogenic influence, etc.) both diversity and faunal composition are indicators of a more or less robust *T. cruzi* transmission cycle in a given area.

**Table 2:** *Trypanosoma cruzi* natural infection in free-living wild mammals from the Amazon Forest biome: taxonomic identification of the examined mammals, results of hemocultures, indirect immunofluorescence tests and characterization of parasites. In the case of insufficient blood for hemocultures and serological tests, we prioritized the blood cultures

Order	Genus	Species	No. of genera (%)^a^	No. of specimens	HC(+)/ specimens tested (%)	IFAT(+)/ specimens tested (%)	DTU (*n*)
Artiodactyla	*Sus*	*Sus scrofa*	11	11	0	8 (73)	
Cingulata	*Dasypus*	*Dasypus novemcinctus*	1/3 (33)	1	1 (100)	nd	TcIV (1)
		*Dasypus* sp*.*		2	0	nd	
	*Euphractus*	*Euphractus sexcinctus*	1	1	0	nd	
Didelphimorphia	*Caluromys*	*Caluromys philander*	2/8 (25)	1	0	1 (100)	
		*Caluromys* sp*.*		7	2 (29)	3 (43)	TcI (1)
	*Didelphis*	*Didelphis albiventris*	24/64 (37)	4	0	0	
		*Didelphis marsupialis*		58	24 (41)	44 (76)	TcI (16); TcII (1); TcI+*T. rangeli* (1); TcI+TcII (2); TcI+TcIII (1); *T. rangeli* (1)
		*Didelphis* sp*.*		2	0	1 (50)	
	*Gracilinanus*	*Gracilinanus* sp*.*	1/17 (6)	17	1 (6)	2 (12)	TcI (1)
	*Marmosa*	*Marmosa murina*	25	7	0	2 (29)	TcI (1)
		*Marmosa* sp*.*		18	0	3 (16)	
	*Marmosops*	*Marmosops parvidens*	1/12 (8)	1	0	1 (100)	
		*Marmosops* sp*.*		11	1 (9)	3 (27)	TcI (1)
	*Metachirus*	*Metachirus nudicaudatus*	6	6	0	0	
	*Micoureus*	*Micoureus demerarae*	1/9 (11)	2	1 (50)	1 (50)	TcI (1)
		*Micoureus* sp*.*		7	0	0	
	*Monodelphis*	*Monodelphis domestica*	2/12 (16)	7	0	1 (14)	
		*Monodelphis* sp*.*		5	2 (40)	2 (40)	TcI (1); TcI+TcIV (1)
	*Philander*	*Philander opossum*	23/67 (34)	57	18 (32)	42 (74)	TcI (14); TcI+TcII (2); *T. rangeli* (1)
		*Philander* sp*.*		10	5 (50)	7 (70)	TcI (3); TcI+TcIII/TcIV (1); TcI+*T. rangeli* (1)
Lagomorpha	*Sylvilagus*	*Sylvilagus brasiliensis*	1	1	0	nd	
Pilosa	*Tamandua*	*Tamandua mirim*	1/2 (50)	2	1 (50)	nd	TcI (1)
Primates	*Alouatta*	*Alouatta belzubul*	3/11 (27)	6	2 (33)	6 (100)	TcI+TcIV (2)
		*Alouatta caraya*		5	1 (20)	2 (40)	TcI+TcIV (1)
	*Sapajus*	*Sapajus libidinosus*	25/46 (54)	46	25 (54)	10 (22)	TcI (2); TcI+*T. rangeli* (10); *T. rangeli* (4)
Rodentia	*Akodon*	*Akodon* sp*.*	1/8 (12)	7	0	0	
		*Akodon lindberghi*		1	1 (100)	0	TcI (1)
	*Cerradomys*	*Cerradomys* sp*.*	1	1	0	1 (100)	
	*Coendou*	*Coendou prehensilis*	2/6 (33)	6	2 (33)	4 (67)	TcI+*T. rangeli* (1)
	*Dasyprocta*	*Dasyprocta prymnolopha*	3	2	0	1 (50)	
		*Dasyprocta* sp*.*		1	0	0	
	*Euryoryzomys*	*Euryoryzomys* sp*.*	1	1	0	0	
	*Holochilus*	*Holochilus* sp*.*	1	1	0	1 (100)	
	*Hylaeamys*	*Hylaeamys megacephalus*	11	4	1 (25)	1 (25)	TcI (1)
		*Hylaeamys* sp*.*		7	0	0	
	*Mus*	*Mus musculus*	1	1	0	0	
	*Necromys*	*Necromys lasiurus*	3	2	0	0	
		*Necromys* sp*.*		1	0	0	
	*Nectomys*	*Nectomys rattus*	6	4	0	0	
		*Nectomys* sp*.*		2	0	0	
	*Oecomys*	*Oecomys* sp*.*	4	4	0	0	
	*Oligoryzomys*	*Oligoryzomys* sp*.*	1	1	0	0	
	*Oryzomys*	*Oryzomys* sp*.*	10	10	0	1 (10)	
	*Oxymycterus*	*Oxymycterus* sp*.*	3	3	0	2 (67)	
	*Proechimys*	*Proechimys roberti*	2/47 (4)	3	0	3 (100)	
		*Proechimys* sp*.*		40	1 (3)	13 (33)	TcI+TcIII/TcIV (1)
		*Proechimys gr. cuvieri*		2	1 (50)	1 (50)	TcI (1)
		*Proechimys gr. goeldii*		1	0	0	
		*Proechimys gr. guianensis*		1	0	1 (100)	
Total: 7	31			401	90/402 (22)	168/398 (42)	*P* <0.0001

^a^Number of genera with infected species/number of genera examined

*Abbreviations*: HC(+), number of specimens with positive hemocultures; IFAT(+), number of specimens with positive IFAT test; DTU, Discrete Typing Units; n, number of isolates; nd, not done

**Table 3:** *Trypanosoma cruzi* natural infection in free-living wild mammals from the Caatinga biome: taxonomic identification of the examined mammals, results of hemocultures, indirect immunofluorescence tests, and characterization of parasites. In the case of insufficient blood for hemocultures and serological tests, we prioritized the blood cultures

Order	Genus	Species	No. genera (%)^a^	No. of specimens	HC(+)/ specimens tested (%)	IFAT(+)/ specimens tested (%)	DTU (*n*)
Artiodactyla	*Pecari*	*Pecari tajacu*	1	1	0	0	
	*Sus*	*Sus scrofa*	1	1	0	0	
Carnivora	*Conepatus*	*Conepatus semistriatus*	3	3	0	0	
	*Cerdocyon*	*Cerdocyon thous*	1	1	0	0	
Cingulata	*Dasypus*	*Dasypus novemcinctus*	1/2 (50)	2	1 (50)	nd	TcIII (1)
	*Euphractus*	*Euphractus sexcinctus*	51	51	0	nd	
Didelphimorphia	*Didelphis*	*Didelphis albiventris*	49/134 (36)	127	49 (39)	67 (53)	TcI (34); TcI+*T. rangeli* (1); TcII+TcV/VI (2); *T. rangeli* (1)
		*Didelphis* sp*.*		7	0	4 (57)	
	*Gracilinanus*	*Gracilinanus agilis*	26	13	0	2 (15)	
		*Gracilinanus* sp*.*		13	0	0	
	*Marmosops*	*Marmosops incanus*	10	10	0	0	
	*Monodelphis*	*Monodelphis domestica*	7/94 (7)	80	6 (8)	9 (11)	TcI (1); TcIV (1); TcI+*T. rangeli* (1)
		*Monodelphis* sp*.*		14	1 (7)	2 (14)	nd
	*Thylamys*	*Thylamys velutinus*	2	1	0	0	
		*Thylamys* sp*.*		1	0	0	
Rodentia	*Calomys*	*Calomys expulsus*	10	9	0	0	
		*Calomys* sp*.*		1	0	0	
	*Galea*	*Galea* sp*ixii*	1/47 (2)	38	1 (3)	0	
		*Galea* sp*.*		9	0	0	
	*Kerodon*	*Kerodon rupestris*	2	2	0	0	
	*Mus*	*Mus musculus*	1/15 (7)	15	1 (7)	1 (7)	nd
	*Necromys*	*Necromys lasiurus*	14	14	0	0	
	*Oligoryzomys*	*Oligoryzomys* sp*.*	8	8	0	1 (13)	
	*Oryzomys*	*Oryzomys aff. subflavus*	13	13	0	0	
	*Proechimys*	*Proechimys* sp*.*	2	2	0	0	
	*Rattus*	*Rattus rattus*	15/71 (21)	71	15 (21)	49 (69)	TcI (10); TcII (1)
	*Rhipidomys*	*Rhipidomys macrurus*	26	24	0	8 (33)	
		*Rhipidomys* sp*.*		2	0	0	
	*Thrichomys*	*Thrichomys laurentius*	25/466 (5)	434	23 (5)	72 (17)	TcV (1);TcI+TcIV (1); TcIV (2)
		*Thrichomys inermis*		15	0	6 (40)	
		*Thrichomys apereoides*		5	1 (20)	2 (40)	nd
		*Thrichomys* sp*.*		12	1 (8)	1 (8)	nd
	*Trinomys*	*Trinomys* sp*.*	1	1	0	0	
	*Wiedomys*	*Wiedomys pyrrhorhinos*	5	3	0	1 (33)	
		*Wiedomys* sp*.*		2	0	0	
Total: 5	24			1005	99/1005 (10)	225/952 (24)	*P* <0.0001

^a^Number of genera with infected species/number of genera examined

*Abbreviations*: HC(+), number of specimens with positive hemocultures; IFAT(+), number of specimens with positive IFAT test; DTU, Discrete Typing Units; n, number of isolates; nd, not done

**Table 4:** *Trypanosoma cruzi* natural infection in free-living wild mammals from the Pantanal biome: taxonomic identification of the examined mammals, results of hemocultures, indirect immunofluorescence tests (IFAT), and characterization of parasites. In the case of insufficient blood for hemocultures and serological tests, we prioritized the blood cultures

Order	Genus	Species	No. of genera (%)^a^	No. of specimens	HC(+)/ specimens tested (%)	IFAT(+)/ specimens tested (%)	DTU (*n*)
Artiodactyla	*Sus*	*Sus scrofa*	1/9 (11)	9	1 (11)	0	TcIII (1)
	*Tayassu*	*Tayassu tajacu*	32	4	0	0	
		*Tayassu pecari*		28	0	0	
Carnivora	*Cerdocyon*	*Cerdocyon thous*	3/114 (3)	96	0	53 (55)	
	*Leopardus*	*Leopardus pardalis*	28	28	0	3 (11)	
	*Nasua*	*Nasua nasua*	49/189 (26)	189	49 (26)	52 (28)	*T. rangeli* (2); TcI (33); TcI+*T. rangeli* (4); TcI+TcII (5); TcII (1); TcII+*T. rangeli* (1)
	*Procyon*	*Procyon cancrivorus*	2/15 (13)	15	2 (13)	9 (60)	TcI (1)
Cingulata	*Cabassous*	*Cabassous unicinctus*	3	3	0	nd	
	*Dasypus*	*Dasypus novemcinctus*	2/2 (100)	2	2 (100)	nd	TcIII (2)
	*Euphractus*	*Euphractus sexcinctus*	1/14 (7)	14	1 (7)	nd	TcIII (1)
	*Priodontes*	*Priodontes maximus*	10	10	0	nd	
Didelphimorphia	*Didelphis*	*Didelphis albiventris*	2	2	0	1 (50)	
	*Gracilinanus*	*Gracilinanus agilis*	4/17 (23)	3	3 (100)	0	TcI (2); TcI+TcII (1)
		*Gracilinanus* sp*.*		14	1 (7)	0	TcI (1)
	*Marmosa*	*Marmosa* sp*.*	1/5 (20)	5	1 (20)	1 (20)	nd
	*Marmosops*	*Marmosops* sp*.*	1	1	0	0	
	*Monodelphis*	*Monodelphis* sp*.*	2/14 (14)	7	1 (14)	1 (14)	nd
		*Monodelphis domestica*		7	1 (14)	0	TcI (1)
	*Philander*	*Philander frenatus*	1/1 (100)	1	1 (100)	0	TcI+TcIV (1)
	*Thylamys*	*Thylamys* sp*.*	2/11 (18)	11	2 (18)	1 (9)	TcI (2)
Rodentia	*Calomys*	*Calomys* sp*.*	6	6	0	0	
	*Cerradomys*	*Cerradomys* sp*.*	10	10	0	0	
	*Clyomys*	*Clyomys laticeps*	39	2	0	2 (100)	
		*Clyomys* sp*.*		37	0	0	
	*Dasyprocta*	*Dasyprocta azarae*	8	3	0	0	
		*Dasyprocta* sp*.*		5	0	2 (40)	
	*Holochilus*	*Holochilus* sp*.*	2	2	0	0	
	*Marmosa*	*Marmosa* sp*.*	1	1	0	0	
	*Oecomys*	*Oecomys mamorae*	9/34 (26)	34	9 (100)	0	TcI (5); TcIV (3)
	*Oryzomys*	*Oryzomys* sp*.*	19	19	0	4 (21)	
	*Thrichomys*	*Thrichomys fosteri*	2/216 (1)	138	2 (1)	3 (2)	TcI (1)//TcIV (1)
		*Thrichomys* sp*.*		78	0	23 (29)	
	*Thylamys*	*Thylamys* sp*.*	7	7	0	0	
Total: 5	27			791	75/791 (9)	155/762 (20)	*P* <0.0001

^a^Number of genera with infected species/number of genera examined

*Abbreviations*: HC(+), number of specimens with positive hemocultures; IFAT(+), number of specimens with positive IFAT test; DTU, Discrete Typing Units; n, number of isolates; nd, not done

**Table 5:** *Trypanosoma cruzi* natural infection in free-living wild mammals from the Cerrado biome: taxonomic identification of the examined mammals, results of hemocultures, indirect immunofluorescence tests (IFAT), and characterization of parasites. In the case of insufficient blood for hemocultures and serological tests, we prioritized the blood cultures

Order	Genus	Species	No. of genera (%)^a^	No. of specimens	HC(+)/ specimens tested (%)	IFAT(+)/ specimens tested (%)	DTU (*n*)
Artiodactyla	*Pecari*	*Pecari tajacu*	1	1	0	0	
Carnivora	*Cerdocyon*	*Cerdocyon thous*	59	59	0	22 (37)	
	*Chrysocyon*	*Chrysocyon brachyurus*	91	91	0	6 (6)	
	*Conepatus*	*Conepatus semistriatus*	2	2	0	nd	
	*Eira*	*Eira barbara*	5	5	0	nd	
	*Leopardus*	*Leopardus pardalis*	2/3 (67)	3	2 (67)	1 (33)	TcI (2)
	*Lontra*	*Lontra longicaudis*	1	1	0	nd	
	*Lutra*	*Lutra longicaudis*	1	1	0	nd	
	*Nasua*	*Nasua nasua*	3	3	0	nd	
	*Puma*	*Puma concolor*	2	2	0	2 (100)	
	*Pseudalopex*	*Pseudalopex vetulus*	3/74 (4)	48	2 (4)	22 (46)	TcIII (2);
Cingulata	*Cabassous*	*Cabassous unicinctus*	2	2	0	nd	
	*Dasypus*	*Dasypus novemcinctus*	2	2	0	nd	
	*Euphractus*	*Euphractus sexcinctus*	5	5	0	nd	
Didelphimorphia	*Caluromys*	*Caluromys philander*	1/10 (10)	10	1 (10)	0	TcI (1)
	*Chironectes*	*Chironectes minimus*	4	4	0	0	
	*Cryptonanus*	*Cryptonanus agricolai*	3	3	0	0	
	*Didelphis*	*Didelphis albiventris*	9/118 (7)	104	9 (9)	30 (29)	TcI (4)
		*Didelphis* sp*.*		14	0	4 (29)	
	*Gracilinanus*	*Gracilinanus agilis*	10/191 (5)	92	2 (2)	5 (5)	TcI (2)
		*Gracilinanus* sp*.*		99	8 (8)	14 (14)	TcI (4); TcI+TcIII/TcIV (3); TcI+TcIV (1)
	*Lutreolina*	*Lutreolina crassicaudata*	1	1	0	0	
	*Marmosa*	*Marmosa murina*	59	38	0	1 (4)	
		*Marmosa* sp*.*		21	0	0	
	*Micoureus*	*Micoureus demerarae*	2/11 (18)	3	0	0	
		*Micoureus travassosi*		4	2 (50)	3 (75)	TcI (2)
		*Micoureus* sp*.*		4	0	0	
	*Monodelphis*	*Monodelphis cf. americana*	4/121 (3)	1	0	0	
		*Monodelphis domestica*		93	2 (2)	1 (1)	TcIII (1)
		*Monodelphis kunsi*		2	0	0	
		*Monodelphis sorex*		1	0	0	
		*Monodelphis* sp*.*		24	2 (8)	0	TcI (2)
	*Philander*	*Philander* sp*.*	2	2	0	1 (50)	
	*Thylamys*	*Thylamys* sp*.*	2	2	0	0	
Pilosa	*Myrmecophaga*	*Myrmecophaga tridactyla*	3	3	0	nd	
	*Tamandua*	*Tamandua tetradactyla*	2	2	0	nd	
Primates	*Alouatta*	*Alouatta caraya*	5	5	0	1 (20)	
	*Callithrix*	*Callithrix penicillata*	3	3	0	1 (33)	
	*Cebus*	*Cebus apella*	35	33	0	9 (27)	
		*Cebus* sp*.*		2	0	1 (50)	
Rodentia	*Akodon*	*Akodon montensis*	2/51 (4)	1	0	0	
		*Akodon* sp*.*		50	2 (4)	0	TcI (2)
	*Brucepattersonius*	*Brucepattersonius* sp*.*	8	8	0	0	
	*Calomys*	*Calomys expulsus*	3/286 (1)	51	0	0	
		*Calomys tener*		39	1 (3)	1 (3)	TcI (1)
		*Calomys* sp*.*		196	2 (1)	6 (3)	TcI (2)
	*Cerradomys*	*Cerradomys maracajuensis*	1/28 (3)	5	0	0	
		*Cerradomys marinhus*		9	0	1 (11)	
		*Cerradomys megacephalus*		1	0	0	
		*Cerradomys scotti*		6	0	0	
		*Cerradomys subflavus*		7	1 (14)	0	TcI (1)
		*Cerradomys* sp*.*		27	2 (7)	1 (4)	TcI (2)
	*Dasyprocta*	*Dasyprocta azarae*	3	2	0	nd	
		*Dasyprocta* sp*.*		1	0	nd	
	*Hydrochoerus*	*Hydrochoerus hydrochaeris*	2	2	0	0	
	*Hylaeamys*	*Hylaeamys megacephalus*	3/108 (3)	51	3 (6)	3 (6)	nd
		*Hylaeamys* sp*.*		57	0	0	
	*Juliomys*	*Juliomys* sp*.*	1	1	0	0	
	*Mus*	*Mus musculus*	1	1	0	0	
	*Necromys*	*Necromys lasiurus*	171	166	0	1 (0.6)	
		*Necromys* sp*.*		5	0	2 (40)	
	*Nectomys*	*Nectomys rattus*	45	28	0	7 (25)	
		*Nectomys squamipes*		3	0	0	
		*Nectomys* sp*.*		13	0	0	
	*Oecomys*	*Oecomys bicolor*	2/94 (2)	27	2 (7)	0	
		*Oecomys concolor*		10	0	0	
		*Oecomys* sp*.*		57	0	3 (5)	
	*Oligoryzomys*	*Oligoryzomys fornesi*	2/175 (1)	16	0	0	
		*Oligoryzomys moojeni*		3	0	0	
		*Oligoryzomys nigripes*		8	0	0	
		*Oligoryzomys* sp*.*		148	2 (1)	3 (2)	TcII (1)
	*Oryzomys*	*Oryzomys subflavus*	42	1	0	0	
		*Oryzomys megacephalus*		15	0	4 (27)	
		*Oryzomys scotti*		2	0	1 (50)	
		*Oryzomys* sp*.*		24	0	3 (13)	
	*Oxymycterus*	*Oxymycterus* sp*.*	9	9	0	1 (11)	
	*Proechimys*	*Proechimys roberti*	42	25	0	1 (4)	
		*Proechimys* sp*.*		17	0	2 (12)	
	*Rattus*	*Rattus norvegicus*	1/15 (7)	2	0	0	
		*Rattus rattus*		13	1 (8)	7 (54)	nd
	*Rhipidomys*	*Rhipidomys cf. mastacalis*	1/26 (4)	9	0	0	
		*Rhipidomys macrurus*		2	0	0	
		*Rhipidomys* sp*.*		15	1 (7)	5 (33)	nd
	*Sooretamys*	*Sooretamys* sp*.*	1	1	0	0	
	*Sphiggurus*	*Sphiggurus* sp*.*	1	1	0	0	
	*Thrichomys*	*Thrichomys aff. inermis*	41	7	0	2 (29)	
		*Thrichomys apereoides*		2	0	0	
		*Thrichomys* sp*.*		32	0	9 (28)	
	*Wiedomys*	*Wiedomys* sp*.*	2	2	0	0	
Total: 7	52			1973	47/1973 (2)	191/1838 (10)	*P* <0.0001

^a^Number of genera with infected species/number of genera examined

*Abbreviations*: HC(+), number of specimens with positive hemocultures; IFAT(+), number of specimens with positive IFAT test; DTU, Discrete Typing Units; n, number of isolates; nd, not done

**Table 6:** *Trypanosoma cruzi* natural infection in free-living wild mammals from the Pampa biome: taxonomic identification of the examined mammals and indirect immunofluorescence tests (IFAT)

Order	Genus	Species	No. of genera (%)	No. of specimens	IFAT(+)/ specimens tested (%)
Carnivora	*Cerdocyon*	*Cerdocyon thous*	2	2	1 (50)
	*Leopardus*	*Leopardus geoffroyi*	1	1	0
	*Pseudalopex*	*Pseudalopex gymnocercus*	4	4	3 (75)
Didelphimorphia	*Didelphis*	*Didelphis albiventris*	26	26	8 (31)
Primates	*Alouatta*	*Alouatta* sp*.*	2	2	nd
Rodentia	*Akodon*	*Akodon* sp*.*	2	2	0
	*Mus*	*Mus musculus*	40	40	0
	*Rattus*	*Rattus rattus*	2	1	1 (100)
	*Scapteromys*	*Scapteromys aquaticus*	28	28	2 (7)
	*Sooretamys*	*Sooretamys angouya*	2	2	0
Total	10	8		108	15/106 (14)

*Abbreviations*: IFAT(+), number of specimens with positive IFAT test; nd, not done

**Table 7:** *Trypanosoma cruzi* natural infection in free-living wild mammals from the Atlantic Forest biome: taxonomic identification of the examined mammals, results of hemocultures, indirect immunofluorescence tests (IFAT), and characterization of parasites. In the case of insufficient blood for hemocultures and serological tests, we prioritized the blood cultures

Order	Genus	Species	No. of genera (%)^a^	No. of specimens	HC(+)/ specimens tested (%)	IFAT(+)/ specimens tested (%)	DTU (*n*)
Carnivora	*Cerdocyon*	*Cerdocyon thous*	1	1	0	0	
	*Galictis*	*Galictis vittata*	1/2 (50)	1	1 (100)	nd	TcIII (1)
		*Galictis cuja*		1	0	nd	
	*Leopardus*	*Leopardus pardalis*	4	2	0	2 (100)	
		*Leopardus tigrinus*		2	0	2 (100)	
	*Procyon*	*Procyon cancrivorus*	2	2	0	0	
	*Puma*	*Puma yagouaroundi*	2	2	0	1 (50)	
Cingulata	*Dasypus*	*Dasypus septemcinctus*	4	1	0	nd	
		*Dasypus novemcinctus*		1	0	nd	
		*Dasypus* sp*.*		2	0	nd	
Didelphimorphia	*Didelphis*	*Didelphis albiventris*	77/409 (19)	44	2 (5)	11 (25)	nd
		*Didelphis aurita*		271	69 (25)	81 (30)	TcI (11); TcII (1); TcIV (1); TcI+TcII (2); TcI+TcII+TcIII/TcIV (1); TcI+TcIV (2);TcIII/TcIV (1); TcI+TcII+*T. rangeli* (1); TcI+*T. rangeli* (1); *T. rangeli* (1);
		*Didelphis* sp*.*		94	6 (6)	19 (20)	nd
	*Gracilinanus*	*Gracilinanus microtarsus*	7	1	0	0	
		*Gracilinanus* sp*.*		6	0	0	
	*Marmosa*	*Marmosa paraguayana*	6	1	0	0	
		*Marmosa* sp*.*		8	0	3 (37)	
	*Marmosops*	*Marmosops incanus*	2/14 (14)	3	0	1 (33)	
		*Marmosops paulensis*		6	1 (17)	1 (17)	TcI (1)
		*Marmosops* sp*.*		5	1 (20)	1 (20)	nd
	*Metachirus*	*Metachirus nudicaudatus*	1/15 (7)	15	1 (8)	3 (23)	nd
	*Micoureus*	*Micoureus paraguayanus*	1/7 (14)	7	1 (14)	0	TcIV (1)
		*Micoureus demerarae*		8	0	3 (37)	
	*Monodelphis*	*Monodelphis americana*	2/27 (7)	2	1 (50)	0	
		*Monodelphis dimidiata*		8	0	0	
		*Monodelphis domestica*		3	1 (33)	0	TcI (1)
		*Monodelphis* sp*.*		14	0	0	
	*Philander*	*Philander frenatus*	22/106 (21)	67	19 (28)	8 (12)	TcI (7); TcII (4)
		*Philander opossum*		2	0	0	
		*Philander* sp*.*		37	3 (8)	7 (19)	TcI (1)//TcIV (1); TcI+*T. rangeli* (1)
Primates	*Callithrix*	*Callithrix jacchus*	2/4 (50)	1	1 (100)	0	TcI (1)
		*Callithrix geoffroy*		2	1 (50)	nd	
		*Callithrix* sp*.*		1	0	0	
	*Cebus*	*Cebus apella*	1/1 (100)	1	1 (100)	0	TcII (1)
	*Leontopithecus*	*Leontopithecus chrysomelas*	91/374 (24)	87	39 (45)	48 (55)	TcI (1);TcII (14)
		*Leontopithecus chrysopygus*		1	1 (100)	0	
		*Leontopithecus rosalia*		286	51 (18)	94 (33)	TcI (4); TcII (26); TcI+TcII (1)
Rodentia	*Abrawayaomy*	*Abrawayaomys* sp*.*	1	1	0	0	
	*Akodon*	*Akodon cursor*	2/529 (0.4)	7	0	0	
		*Akodon montensis*		57	0	0	
		*Akodon paranaensis*		7	0	0	
		*Akodon* sp*.*		458	2 (0.5)	20 (4)	TcI (1)
	*Brucepattersonius*	*Brucepattersonius* sp*.*	24	24	0	0	
	*Calomys*	*Calomys* sp*.*	4	4	0	1 (25)	
	*Cavia*	*Cavia* sp*.*	2	2	0	1 (50)	
	*Cerradomys*	*Cerradomys* sp*.*	1	1	0	0	
	*Coendou*	*Coendou* sp*.*	1	1	0	0	
	*Delomys*	*Delomys dorsalis*	1	1	0	0	
	*Euryoryzomys*	*Euryoryzomys russatus*	9	9	0	0	
	*Euryzygomatomys*	*Euryzygomatomys spinosus*	3	3	0	0	
	*Galea*	*Galea* sp*ixii*	1	1	0	0	
	*Hyleamys*	*Hyleamys* sp*.*	1	1	0	0	
	*Juliomys*	*Juliomys pictipes*	1	1	0	0	
	*Mus*	*Mus musculus*	16	15	0	0	
		*Mus* sp*.*		1	0	0	
	*Necromys*	*Necromys lasiurus*	12	12	0	2 (16)	
	*Nectomys*	*Nectomys squamipes*	3/135 (2)	30	1 (3)	4 (13)	TcI (1)
		*Nectomys rattus*		6	0	1 (17)	
		*Nectomys* sp*.*		99	2 (2)	1 (1)	TcI (1)
	*Oligoryzomys*	*Oligoryzomys flavescens*	479	1	0	0	
		*Oligoryzomys nigripes*		116	0	3 (3)	
		*Oligoryzomys* sp*.*		362	0	1	
	*Oryzomys*	*Oryzomys* sp*.*	4	4	0	0	
	*Oxymycterus*	*Oxymycterus judex*	32	14	0	0	
		*Oxymycterus* sp*.*		18	0	3 (17)	
	*Rattus*	*Rattus rattus*	35	35	0	9 (26)	
	*Rhipidomys*	*Rhipidomys* sp*.*	3	3	0	1 (33)	
	*Sooretamys*	*Sooretamys angouya*	12	12	0	0	
	*Sphiggurus*	*Sphiggurus villosus*	1	1	0	0	
	*Thaptomys*	*Thaptomys nigrita*	81	81	0	0	
	*Thrichomys*	*Thrichomys apereoides*	8	6	0	2 (33)	
		*Thrichomys* sp*.*		2	0	0	
	*Trinomys*	*Trinomys iheringi*	24	3	0	0	
		*Trinomys paratus*		10	0	2 (20)	
		*Trinomys* sp*.*		11	0	4 (36)	
Total: 5	43			2416	205/2416 (8.5)	340/2375 (14)	*P* <0.0001

^a^Number of genera with infected species/number of genera examined

*Abbreviations*: HC(+), number of specimens with positive hemocultures; IFAT(+), number of specimens with positive IFAT test; DTU, Discrete Typing Units; n, number of isolates; nd, not done

The *T. cruzi* infection rate of free-living mammals in the Amazon biome was significantly higher than observed in other biomes, both in relation to blood cultures and serology (IFAT). Free living mammals of Caatinga, Pantanal and Atlantic forest displayed comparable rates of positive hemocultures (Table 1; *P* < 0.0001). Moreover, the distribution of *T. cruzi* DTUs in Brazilian biomes is apparently non-homogeneous. As an example, the DTU TcII that was rarely found in the Amazon and even then, in localities that are distant from each other (Table 2). This observation raises the question of how is a *T. cruzi* DTU that is only sporadically isolated, maintained and dispersed in the natural environment? Actually, there is still much to learn about the ecology of *T. cruzi* and its DTUs.

In the Cerrado, most trapped and examined mammals were rodents (54.6%) (Table 5). Primates accounted for only 2.3% of the examined mammals, while marsupials and carnivores constituted 27.4 and 15.6% of sampled animals, respectively (Table 5). Only 2% of all examined mammals displayed positive hemocultures in this biome, which was statiscally different from other biomes (Table 1; *P* < 0.0001).

It is worth noting that although the number of examined mammals and study areas is large, these are still not representative considering the huge diversity and number of the extant mammalian fauna and the huge ecological diversity and dimensions of the country. It was not possible for our group to test all of the examined wild mammal species by serological methods due to the lack of species-specific conjugates and/or insufficient amount of serum. Actually, blood cannot be collected from very small-sized animals in sufficient quantities for blood cultures (one pair of tubes with 300 μl each) and serology (50 μl sera).

## *Trypanosoma cruzi* transmission among mammals with low parasitemia

In the Pampa biome, 14% of the animals demonstrated positive serology but none displayed positive blood culture (Table 6). The questions that immediately arise are: how is transmission possible as it depends on high parasitaemia, and what is the strategy that ensures the permanence of the parasite in this scenario? In contrast, in the Amazon, 22% of mammals including those using all forest strata presented high parasitaemia expressed by positive blood cultures in a classical epizootic scenario (Table 2). A hypothesis we favor is that the mammal fauna in the Pampa display only short periods of high parasitaemia, i.e. the transmission window of these individuals is narrow, and it is the assemblage of mammals at different stages of infection that ensures the persistence of *T. cruzi* in nature [2]. In areas in which mammalian fauna display low parasitemia, the transmission of *T. cruzi* may also rely on ecological features of hosts and triatomine, e.g. in higher host-vector contact as is the case of nesting animal species such as coatis, hoary foxes and golden lion tamarins.

The overall low *T. cruzi* infection rates observed in the order Rodentia that are highly susceptible to experimental infection suggest that these mammals play a secondary role as reservoirs in the wild (Tables 2, 3, 4, 5, 6, 7 and 8). This was especially striking in the Cerrado biome, as expressed by their low infection rates (3.7% of positive serological tests and 0.7% of positive hemocultures) (Table 5). Two hypotheses may explain these findings: (i) the infected animals do not resist the infection and die; or (ii) rodents are not exposed to infection in the studied Cerrado regions. Finally, these findings are similar to what was observed in the Pantanal biome [15] and support the hypothesis that, in fact, wild rodents only exceptionally play an important role as *T. cruzi* reservoirs.

**Table 8:** *Trypanosoma cruzi* natural infection in free-living wild mammals of Brazilian biomes: mammalian taxa that present the highest competencies for infection as expressed by positive blood cultures

Order	Genus	Biome	No. of specimens	HC(+)/ specimens tested (%)	IFAT(+)/ specimens tested (%)	*P-*value
Didelphimorphia	*Didelphis*	Amazon Forest	64	24 (37)	45 (70)	0.0001
	*Philander*		68	24 (35)	50 (73)	<0.0001
	*Didelphis*	Atlantic Forest	410	77 (19)	111 (27)	0.004
	*Philander*		106	22 (20)	15 (14)	0.2776
	*Didelphis*	Caatinga	134	49 (36)	71 (53)	0.0099
	*Didelphis*	Cerrado	118	9 (8)	34 (29)	<0.0001
Primates	*Leontopithecus*	Atlantic Forest	374	91 (24)	205 (55)	<0.0001
	*Sapajus*	Amazon Forest	46	25 (54)	10 (22)	0.0026
	*Alouatta*		11	3 (27)	8 (73)	0.0881
Carnivora	*Nasua*	Pantanal	230	76 (33)	77 (33)	0.9212

*Abbreviations*: HC(+), number of specimens with positive hemocultures; IFAT(+), number of specimens with positive indirect immunofluorescence test (IFAT)

The miscellaneous, focal and aggregated character of the *T. cruzi* transmission cycle in the wild environment can be observed in two biomes. In the Atlantic Forest, of the 8.5% of the mammals showing positive blood cultures, 44.4% were constituted by two species of tamarins: 37.6% by *Didelphis* spp. and 10.7% by *Philander* spp. Overall, in the Atlantic Forest, only 0.35% of the rodents displayed positive blood cultures (Table 7). In the Caatinga, we observed one rodent species (*Rattus rattus*) with high rates of positive blood cultures (21%, Table 3), showing how important it is to look at every forest fragment as a singularity. Transmission patterns may change in time as revealed by studies of *Trypanosoma* spp. infection in coatis of a Pantanal region termed Nhecolandia. Initially, high rates of infection with *T. evansi* were observed (Table 4) [35]. The continued monitoring of this same coati population resulted in successful isolations of *T. cruzi* and also *T. rangeli* [30]. More recently, coatis demonstrated to be again infected with *T. evansi* and to a lesser extent with *T. cruzi* or *T. rangeli* [2]. It is tempting to hypotethize that climatic alterations resulted in this process, but which ones? *Trypanosoma evansi* and *T. cruzi* display distinct transmission strategies *T. evansi* being mechanically transmitted by hematophagous dipterans and *T. cruzi* and *T. rangeli*, by triatomine vectors. All these data together show that host-parasite systems are complex and unpredictable.

An example of independent transmission cycles occurring in the same forest fragment was observed in a rainforest fragment in the Atlantic Forest biome where a conservation program of the endangered golden lion tamarin (GLT) species, *Leontophithecus rosalia*, is maintained [25]. The study of *T. cruzi* infection of GLTs and sympatric mammals revealed that only GLTs were infected with *T. cruzi* DTU TcII and all other species of local mammals showed infections with TcI (Table 7) [25].

Of the examined infected mammals, those species that demonstrated the higher infectivity potential, as expressed by high rates of positive hemocultures throughout Brazilian biomes will be highlighted here. These mammal taxa are Didelphimorphia (*Didelphis* spp*.* and *Philander* spp*.*), procyonid carnivores (*N. nasua*) and primates (Table 8). It is worth mentioning that representatives of the Didelphimorphia displayed the highest infective competence in the Amazon, Atlantic Forest and Caatinga (along with primates in the first two biomes). In the Pantanal, *N. nasua* plays this role. Bats were distinguished for presenting the highest diversity of *Trypanosoma* spp. infection in simple and mixed infections and are discussed separately (Table 9).

**Table 9:** *Trypanosoma* spp. natural infection in Chiroptera from Brazilian biomes: partial results of characterization of *Trypanosoma* spp. isolates derived from 1218 bats collected in five Brazilian biomes Parasite molecular target (V7V8 SSU rRNA / Cytb / 18S)

Biome	Genus	Species	No. of genera (%)^a^	No. of specimens	HC(+)/ specimens tested (%)	*Trypanosoma* spp. or *T. cruzi* DTU (*n*)
Amazon Forest	*Anoura*	*Anoura caudifer*	1	2	1 (50)	*T. cruzi* TcI (1)
	*Artibeus*	*Artibeus lituratus*	127 (15.7)	59	6 (10)	*Trypanosona* sp. (1); *T. cruzi* TcIV (2); *T. cruzi* TcI (3)
		*Artibeus* cf. *fimbriatus*		1	1 (100)	*T. cruzi* TcI (1)
		*Artibeus obscurus*		5	1 (20)	*Trypanosona* sp. (1)
		*Artibeus planirostris*		62	12 (19)	*Trypanosona* sp*.* (4); *T. c. marinkellei* (2); *T. cruzi* TcI (4); *T. rangeli* (sub-população A) (1); *T. dionisii* (1)
	*Carollia*	*Carollia perspicillata*	131 (25.1)	118	29 (24)	*T. cruzi* TcI (20); *T. dionisii* (3); *T. rangeli* (subpopulation A) (1); *Trypanosona* sp. (4); *Trypanosoma* sp. + *T. cruzi* TcI (1)
		*Carollia* cf. *beikeith*		1	1 (100)	*T. cruzi* TcI (1)
		*Carollia brevicauda*		12	3 (25)	*T. cruzi* TcI (3)
	*Chiroderma*	*Chiroderma villosum*	3	3	0	
	*Dermanura*	*Dermanura cinereus*	13 (7.0)	13	1 (7)	*T. cruzi* TcI (1)
	*Desmodus*	*Desmodus rotundus*	2	2	0	
	*Diaemus*	*Diaemus youngi*	1	1	0	
	*Eptesicus*	*Eptesicus brasiliensis*	3 (33.3)	3	1 (33)	*Trypanosona* sp. (1)
	*Glossophaga*	*Glossophaga soricina*	11 (18.2)	11	2 (18)	*T. cruzi* TcIV (1); *T. cruzi* TcI (1)
	*Lasiurus*	*Lasiurus blossevillii*	2 (50.0)	2	1 (50)	*T. cruzi* TcI (1)
	*Lonchophylla*	*Lonchophylla thomasi*	5 (20.0)	5	1 (20)	*T. cruzi* TcI (1)
	*Lophostoma*	*Lophostoma silvicolum*	3 (66.7)	3	2 (66)	*T. c. marinkellei* (2)
	*Mesophylla*	*Mesophylla macconelli*	2	2	0	
	*Micronycteris*	*Micronycteris megalotis*	1	1	0	
	*Molossus*	*Molossus aff. rufus*	3	3	0	
	*Noctilio*	*Noctilio albiventris*	1	1	0	
	*Mimom*	*Mimom crenulatum*		1	0	
	*Phyllostomus*	*Phyllostomus discolor*	32 (53.1)	7	6 (85)	*T. cruzi* TcI (2); *T. c. marinkellei* (2); *Trypanosona* sp. (2)
		*Phyllostomus elongatus*		12	2 (16)	*Trypanosona* sp. (1); *T. c. marinkellei* (1)
		*Phyllostomus hastatus*		13	9 (69)	*Trypanosona* sp. (3); *T. c. marinkellei* (5); *T. cruzi* TcIV + *T. c. marinkellei* (1)
	*Plathyrhinus*	*Plathyrhinus infuscus*	3 (33.3)	2	1 (50)	*T. cruzi* TcI (1)
		*Plathyrhinus incarum*		1	0	
	*Rhynophylla*	*Rhynophylla pumilio*	9	5	0	
		*Rhinophylla fischerae*		4	0	
	*Saccopteryx*	*Saccopteryx leptura*	1	1	0	
	*Sturnira*	*Sturnira lilium*	6 (33.3)	2	1 (50)	*Trypanosona* sp. (1)
		*Sturnira tildae*		4	1 (25)	*T. dionisii* (1)
	*Tonatia*	*Tonatia saurophila*	3 (33.3)	3	1 (33)	*T. cruzi* TcI (1)
	*Trachops*	*Trachops cirrhosus*	3 (100)	3	3 (100)	*T. cruzi* TcI (2); *T. dionisii* (1)
	*Uroderma*	*Uroderma bilobatum*	13 (7)	13	1 (7)	*T. cruzi* TcI (1)
	*Vampyrum*	*Vampyrum spectrum*	1	1	0	
	*Vampyrodes*	*Vampyrodes caraccioli*	1	1	0	
	*Vampyressa* sp*.*	*Vampyressa* sp.	1 (100)	1	1 (100)	*T. cruzi* TcI (1)
Atlantic Forest	*Ametrida*	*Ametrida centurio*	1	1	0	
	*Anoura*	*Anoura caudifer*	21 (14.2)	11	1 (9)	*Trypanosona* sp. (1)
		*Anoura geoffroyi*		10	2 (20)	*T. dionisii* (2)
	*Artibeus*	*Artibeus cinereus*	305 (1.3)	21	0	
		*Artibeus fimbriatus*		6	1 (16)	*T. cruzi* TcI (1)
		*Artibeus lituratus*		81	2 (2)	*T. cruzi* TcIII (1); *T. cruzi* TcIII/V (1)
		*Artibeus obscurus*		4	0	
		*Artibeus planirostris*		193	1 (0.5)	*T. cruzi* TcI (1)
	*Carollia*	*Carollia perspicillata*	133 (18.0)	133	24 (18)	*T. cruzi* TcIV (1); *T. dionisii* (20); *Trypanosoma* sp. (1); *Trypanosoma rangeli* D (1); *Trypanosoma rangeli* B (1)
	*Chrotopterus*	*Chrotopterus auritus*	1	1	0	
	*Dermanura*	*Dermanura cinerea*	22	22	0	
	*Desmodus*	*Desmodus rotundus*	30 (16.7)	30	5 (16)	*T. cruzi* TcIII/V (1); *T. cruzi* TcI (1)*; Trypanosoma* sp. (1); *T. dionisii* (2)
	*Lonchorrina*	*Lonchorrina aurita*	1	1	0	
	*Glossophaga*	*Glossophaga soricina*	23 (4.3)	23	1 (4)	*T. dionisii* (1)
	*Glyphonycteris*	*Glyphonycteris sylvestris*	1	1	0	
	*Lophostoma*	*Lophostoma silvicolum*	1	1	0	
	*Micronycteris*	*Micronycteris microtis*	1/3 (33.3)	2	0	
		*Micronycteris minuta*		1	1 (100)	*T. cruzi* TcI/III (1)
	*Mimon*	*Mimon bennettii*	2	2	0	
	*Myotis*	*Myotis nigricans*	10 (40.0)	10	4 (40)	*T. dionisii* (3); *T. cruzi* TcIII/V (1)
	*Phyllostomus*	*Phyllostomus discolor*	23 (13.0)	11	3 (27)	*T. cruzi* TcI (1); *T. c. marinkellei* (2)
		*Phyllostomus hastatus*		12	0	
	*Platyrrhinus*	*Platyrrhinus lineatus*	9	9	0	
		*Platyrrhinus recifinus*		1	0	
	*Rinophyla*	*Rinophyla pumilio*	5	5	0	
		*Rinophyla* sp.	1 (5.9)	1	0	
	*Sturnira*	*Sturnira lilium*	41 (12.1)	40	5 (12)	*T. dionisii* (4); *Trypanosona* sp. (1)
		*Sturnira tildae*		1	0	
	*Tonatia*	*Tonatia bidens*	3 (33.3)	3	1 (33)	*T. dionisii* (1)
	*Tracops*	*Tracops cirrhosus*	1 (100)	1	1 (100)	*Trypanosoma* sp. (1)
	*Vampyressa*	*Vampyressa pusilla*	1	1	0	
Caatinga	*Phyllostomus*	*Phyllostomus* sp.	2	2	0	
	*Molossus*	*Molossus* sp.	3	3	0	
Cerrado	*Artibeus*	*Artibeus lituratus*	49 (10.2)	11	1 (9)	*Trypanosoma* sp. (1)
		*Artibeus planirostris*		37	4 (10)	*Trypanosoma* sp. (3); *T. c. marinkellei* (1)
		*Artibeusaff. Obscurus*		1	0	
	*Carollia*	*Carollia perspicillata*	24 (4.0)	24	1 (4)	*Trypanosoma* sp. (1)
	*Desmodus*	*Desmodus rotundus*	4 (25.0)	4	1 (25)	*Trypanosoma* sp. (1)
	*Glossophaga*	*Glossophaga soricina*	7 (14.0)	7	1 (14)	*Trypanosoma* sp. (1)
	*Molossops*	*Molossops* sp.	2	2	0	
	*Phyllostomus*	*Phyllostomus albicola*	12 (83.3)	1	1 (100)	*Trypanosoma* sp. (1)
		*Phyllostomus discolor*		1	1	*Trypanosoma* sp. (1)
		*Phyllostomus hastatus*		11	9 (81)	*Trypanosoma* sp. (4)
	*Platyrhinus*	*Platyrhinus lineatus*	7	7	0	
	*Sturnira*	*Sturnira lilium*	4 (25.0)	4	1 (25)	*T. dionisii* (1)
	*Eptesicus*	*Eptesicus brasiliensis*	3	3	0	
	*Eumops*	*Eumops* sp.	6	6	0	
	*Myotis*	*Myotis nigricans*	1	1	0	
	*Rhynchonycteris*	*Rhynchonycteris naso*	1 (5.9)	1	0	
Pantanal	*Artibeus*	*Artibeus jamaicensis*	16 (18.7)	1	1 (100)	*Trypanosoma* sp. (1)
		*Artibeus planirostris*		15	2 (13)	*Trypanosoma* sp. (1); *T. c. marinkellei* (1)
	*Carollia*	*Carollia perspicillata*	4 (5.9)	4	0	
	*Chrotopterus*	*Chrotopterus autirus*	1 (5.9)	1	0	
	*Desmodus*	*Desmodus rotundus*	18 (16.6)	18	3 (16)	*T. c. marinkellei* (1); *Trypanosoma* sp. (2)
	*Glossophaga*	*Glossophaga soricina*	3	3	0	
	*Lophostoma*	*Lophostoma silvicolum*	5	5	0	
	*Molossops*	*Molossops temminckii*	1	1	0	
	*Molossus*	*Molossus molossus*	2	2	0	
	*Myotis*	*Myotis migricans*	1	1	0	
	*Noctilio*	*Noctilio albiventris*	2	2	0	
	*Platyrrhinus*	*Platyrrhinus lineatus*	9	9	0	
	*Phyllostomus*	*Phyllostomus hastatus*	5 (60.0)	5	3 (60)	*Trypanosoma* sp. (1); *T. c. marinkellei* (2)
	*Sturnira*	*Sturnira lilium*	3 (33.3)	3	1 (33)	*T. dionisii* (1)
Total	76	94	1218 (5.9)	1219	168 (14)	

^a^Number of genera with infected species/number of genera examined

*Abbreviations*: HC(+), number of specimens with positive hemocultures; DTU, Discrete Typing Units; n, number of isolates

## Didelphimorphia (*Didelphis* spp. and *Philander* spp.)

Marsupials comprise a mammal group which presents a peculiar reproduction strategy. These ancient mammals are an evolutionary success since they are extant, with little anatomical change since the Cretaceous [36]. The family Didelphidae includes all representatives of metatherian mammals or marsupials extant in the Americas. Didelphidae includes four subfamilies (Glironiinae, Caluromyniae, Hyladelphinae and Didelphinae), 19 genera and 95 species of which only four do not occur in South America [37].

The species within the genus *Didelphis* include the largest American marsupials. *Didelphis* spp. have a pair of scent glands adjacent to the cloaca that are a suitable niche for the multiplication of *T. cruzi* as epimastigote forms and for its differentiation to infective metacyclic trypomastigotes [4]. Didelphid marsupials have a very short gestation time (in Didelphidae, delivery occurs on average after 13 days of gestation). Individuals are born in an almost embryonic stage; the newborns do not have obvious eyes, ears or hind legs. They are absolutely hairless, and their mouth is a small hole through which the newborn will adhere to a nipple, and to which it will remain attached and totally dependent for six to eight weeks. Nevertheless, the newborn marsupial must climb from the womb without maternal aid to reach the pouch and adhere to a nipple. At this stage, and for the next several weeks, the newborn is totally dependent on the marsupium, and if removed will not survive. Despite the close and long contact between the newborn with the mother, in experimental conditions, we never observed neonatal transmission of *T. cruzi* in *D. aurita* [38]*.*

Often described as essentially arboreal, *Didelphis* spp*.* use with reasonable frequency the soil, the understory, and the sub-canopy/canopy strata. However, the use of vertical space is not a fixed trait; it depends on the population structure, in that competition between young and adult animals may determine the level of occupation of the vertical space. Interspecific competition may also determine the use of the vertical space by this taxon [39]. In the wild, *Didelphis* spp*.* also use tree hollows, wood piles, palm crown and other locations for shelter, all of which are excellent habitats for triatomine species. Didelphidae are classically described as solitary animals with social interactions restricted to the mating season and to young pouch animals. However, this paradigm is being challenged by recent observations suggesting that these animals may display a social behavior pattern [40]. Didelphid marsupials are nocturnal, nomadic (mainly the males) and are agile climbers but quite clumsy when on the ground. *Didelphis* spp. can become infected orally (preying on small mammals or triatomines); they display high infectivity potential mainly for TcI (with high levels of parasitaemia almost indefinitely) and are able to infect other mammals directly in antagonistic situations as *T. cruzi* could be present in the secretions from the scent glands that are released by stress.

*Trypanosoma cruzi* is not homogeneously distributed in the lumen of the scent glands. The epimastigotes are found along the glandular epithelium that is coated by hyaluronic acid. The metacyclic forms are found mainly in the middle of the lumen of the glands and metacyclogenesis occurs regardless of adhesion [41]. This ensures the preferential elimination of infectious trypomastigotes when scent glands are used. All these characteristics have, as a consequence, that didelphids are exposed to all of the *T. cruzi* transmission cycles that occur in these habitats.

*Didelphis* spp*.* are extremely habitat and food resilient and easily adapt to peri-domestic areas. There, they feed on human food remains and also serve as food for humans. This is especially the case among the riverine settlements in the Amazon rainforest. Besides being an important source of protein, *Didelphis* spp. are also used for medicinal purposes by these populations; the meat is described as tasty and of good quality [42]. The preparation of opossums without the proper care of hygiene also represents a *T. cruzi* infection risk to humans.

Marsupials of the genus *Philander* are smaller than *Didelphis* spp. *Philander* spp. are extremely agile on the ground and also display remarkable climbing ability. They are also described as the most aggressive didelphids. These opossums are exclusively nocturnal and, although often caught on the soil, they are also arboreal. They live in dense forests or underbrush where they build their nests of leaves but may also eventually use branches or fallen trunks as shelter. Their preferred environments appear to be gallery forests or other more humid environmetns, where they are often found. *Philander* spp. display quite eclectic feeding habits and predate both on insects and small vertebrates, including mammals. They also feed on fruits and eggs.

Due to their nomadic character, generalist feeding and housing habits, *Philander* spp*.* and *Didelphis* spp*.* are exposed to *T. cruzi* transmission cycles occurring in all forest strata in the wild. Interestingly, species of these two genera of marsupials handle experimental infections with *T. cruzi* differently. Thus, *Didelphis* spp. control to subpatent levels infections with the Y strain of *T. cruzi* (TcII) but maintain indefinitely high parasitaemias (expressed by positive blood cultures) in infections with TcI strains. In contrast, *Philander* spp. maintain high parasitaemia when inoculated with strain Y (TcII) and control the parasitemia with TcI strains [43]. TcI and TcII are, however, found in mixed infections, and this is the most common and most widely dispersed combination that we found infecting both mammals and vectors [2].

As shown in Table 8, *Didelphis* spp*.* and *Philander* spp*.* demonstrated high rates of positive blood cultures in the Atlantic Forest and the Amazon. In the semi-arid region of the north-eastern Brazil. *Didelphis* spp*.* act as an important reservoir. In the Pantanal, in addition to being uninfected, the relative abundance of these two species was very low. In this immense flood plain region, the primary reservoir of *T. cruzi* is the procyonid *N. nasua*.

A very interesting and puzzling finding was reported by Dario et al. [44] who observed one *Monodelphis americana* (Didelphimorphia) specimen infected simultaneously with *T*. *cascavelli*, *T*. *dionisii* and *Trypanosoma* sp. *Trypanosoma cascavelli* was originally described infecting a *Crotalus durissus terrificus* [45]. There are three interesting aspects here: (i) the infection of a marsupial with a *Trypanosoma* species associated exclusively to bats (*T. dionisii*); (ii) a *Trypanosoma* species that is able to infect both a mammal and a reptile in a mixed infection with *T. dionisii*; and (iii) the presence of a still unknown taxonomic unit of *Trypanosoma* sp. Concerning the second item, Didelphimorphia display lower temperatures in comparison to other mammalian taxa, a feature that could enhance the adaptation chances of *T. cascavelli* to this mammal taxon*.* Moreover, transmission may be due to a hematophagous insect that feeds on both marsupials and snakes, or due to predation of *Monodelphis* spp. by *Crotalus* sp. Actually, *Monodelphis* spp. are small-sized marsupials that definitively are not able to feed on rattlesnakes. These findings were only possible because Dario et al. used NGS since *T. cascavelli* is not cultivable in axenic media. As such, it is clear that we are still underestimating the diversity of *Trypanosoma* spp. in marsupials.

The infection of marsupials with *T. dionisii* reinforces this species as a generalist species, since this parasite was already found infecting humans [18]. Actually, humans, bats and marsupials are phylogenetically very distant taxa.

## The coati, *N. nasua*, and other carnivores

Working with carnivores is not easy and requires a complex infrastructure and well-trained experienced professionals. This might explain why most studies of reservoirs of *T. cruzi* do not include representatives of this group. Most carnivores are adapted to terrestrial life, but they may also be excellent swimmers and climbers. Carnivores use large living areas, are at the top of the food chain and are talented hunters. Consequently, it is very probable that these animals are more prone to infection *via* the oral route. Not all carnivores are flesh eaters *sensu stricto*. There are opportunistic carnivore species that also feed on insects and fruits. Only the so-called hypercarnivore (majority of the felids) depend almost exclusively on flesh.

All carnivores, including domestic dogs and cats, may be involved in the transmission cycle of *T. cruzi* [32, 33, 46]. Concerning wild carnivores, infection with *T. cruzi* was demonstrated in 15 Brazilian Neotropical wild carnivore species [3]. A link between diet peculiarities and *T. cruzi* infection rates in these taxa could also be observed. Thus, insectivore canids and hypercarnivores (those that include flesh as more than 70% of their diet) displayed higher *T. cruzi* infection rates than other carnivores with other types of diet. These findings confirmed that *T. cruzi* transmission cycles are deeply immersed in the trophic nets. Additionally, differences in infectivity potential across the carnivore species have also been described the Procyonids, mainly *N. nasua*, *N. narica* and *Procyon lotor*, being the carnivore species that display the more expressive infectiveness potential as expressed by high and long-lasting parasitemias [3, 27, 47–49]. Note that Procyonids are omnivores that also feed on both insects and small mammals [50], a trait that enhances their chances of infection with the diverse *T. cruzi* DTUs.

Coatis are scansorial, diurnal and generalist procyonid carnivores that use both the arboreal and terrestrial strata [50, 51]. They are peculiar mammals in that they construct and use arboreal nests for resting and birthing [52]. One individual may use several nests during its lifespan, or more than one individual or even a whole social group may share the same resting nest. This behavior increases the probability of encounter between coatis and triatomines, since bugs also use these nests as shelter, consequently enhancing *T. cruzi* transmission chances. *Nasua nasua* are organized in social groups that include mainly females and young, still immature individuals, and only a few adult males [53]. The birthing nests are constructed by the females that give birth to up to seven pups. In these nests, which are larger than the resting nests, the female remains caring for her cubs for about two months [51, 52].

Encounter rates between free-ranging mammals and vectors are one of the most important and challenging variables to be determined. Actually, animal shelters and nests may be used successively, by several species of living organisms, including triatomine vectors of *T. cruzi*. Coatis arboreal nests offer shelter for several other animal taxa, forming genuine and complex biocenoses. On dissection, these nests can be found to contain ants, lizards, beetles and triatomines. Arboreal nests of coatis have also been demonstrated to be suitable habitats for *Rhodnius stalii* and *Triatoma sordida*, which were described living in sympatry, in a same arboreal coati’s nest. Even a terrestrial rodent has been described visiting a coati’s nest [54]. Probably, this rodent used fallen trunks or vines to reach the nest that was over ten meters above the ground. This report shows that the detection of a particular *T cruzi* DTU in an individual mammalian host species does not necessarily mean that there is an association between the DTU and that species or forest stratum.

During a study of coati ecology, Santos et al. [55] collected *Triatoma sordida* (*n* = 13) and *Rhodnius stali* (*n* = 2) from seven coati resting nests. Additionally, 21 nymphs of all stages were collected from these nests, confirming these as suitable habitats for triatomines. Seventeen isolates of *T. cruzi* derived from insects of four coati nests were obtained, mostly TcI. One single insect was found infected with TcIII, and four individuals displayed mixed infections with TcI and TcIII. The use of these nests by coatis and other animals provides a suitable habitat for the maintenance of the triatome colonies since sufficient food source and shelter is offered.

As avid insect predators [50], coatis may acquire *T. cruzi* infections *via* the oral route, which is recognized as highly efficient [56]. In fact, the dense fur of the majority of wild mammal species most probably acts as a barrier against the infection *via* the contaminative route. *Nasua nasua* has been described as a competent *T. cruzi* reservoir due to their long-lasting parasitemia demonstrated by positive hemocultures. Furthermore, *N. nasua* has been shown to be able to maintain the main genotypes of *T. cruzi* (TcI and TcII) in mixed or single infections (Table 4). This ability to maintain *T. cruzi* populations, together with their generalist character, suggests that the coatis may be exposed to many or all of the various transmission cycles of the parasite. Taken all together, this implies that this species may act as a hub of the *T. cruzi* transmission network and a bio-accumulator of *T. cruzi* subpopulations [3, 27, 30, 47]. Also of interest is that Rocha et al. [3] observed *T. cruzi* isolates that did not fit into any of the six recognized DTUs, suggesting that there is still much to learn about the diversity of this parasite. Table 4 shows infection rates and *T. cruzi* genotypes observed in *N. nasua* from Pantanal region.

## Primates: golden lion tamarins in the Atlantic Forest and Cebidae in the Amazon region

New World primates are essentially arboreal, and some use their prehensile tail as a fifth limb. It is currently accepted that this taxon (as well as caviomorph rodents) have entered the Americas coming from Africa after crossing the Atlantic Ocean 35 MYBP [57, 58]. Very probably, they were included in the *T. cruzi* transmission cycle maintained at that time by the autochthonous mammalian fauna once they arrived the New World.

Primates of distinct genera and species may act as important reservoirs of *T. cruzi* (Table 8) with positive hemocultures in 119 of 431 (27.6%) primates examined. These primates were infected with TcI and TcII (Tables 2 and 7). Tamarins (*Leontopithecus rosalia* and *L. chrysomelas*) were found infected with *T. cruzi* in the Atlantic Costal Forest in both the southeast and the northeast regions, mainly with TcII, but also with TcI (Table 7).

Tamarins are diurnal primates that live in social groups of up to ten individuals. *Leontopithecus rosalia*, the golden lion tamarin (GLT), is an elegant and delicate primate; the adult weighs between 360 and 710 g, and 60 g is considered normal for a GLT puppy. They are endemic to the Atlantic Forest and are in serious danger of extinction. Tamarins can live up to 15 years and are essentially arboreal.

A ten-year follow-up of *T. cruzi* infection revealed that *L. rosalia* is able to maintain a stable and long-lasting *T. cruzi* TcII transmission focus. It is interesting to note that in the studied area, only *L. rosalia* showed infection with this DTU: the other mammals were infected only with TcI. *Leontopithecus rosalia* infection with TcI was also observed in the area that is made up of a complex of forest fragments designed for tamarin conservation. Still in the Atlantic Forest, but in the northeast of the country, another species of tamarin (*L. chrysomelas*) also showed high rates of infection with *T. cruzi* TcII [25]. Such a stable transmission cycle of *T. cruzi* DTU TcII is not frequently found in the wild environment. What is seen in nature is that TcII infects numerous species of wild mammals distributed in all biomes but at low rates of infection. *Trypanosoma cruzi* DTU TcI is strongly predominant among free-living wild mammals [2]. An interesting aspect shown by the study of *T. cruzi* infection in tamarins is that even in a small area (5500 ha), such as the forest fragment where these primates were studied, the *T. cruzi* infection distribution was aggregated [25].

The congenital infection of *L. rosalia* is probably not very frequent because *T. cruzi* infection was not observed in juveniles [25]. Possible sites of infection are the hollows of trees that serve as a night shelter for these animals. Each hollow is shared by a social group in a rotation system of the hollows used. Inside a hollow, animals sleep tightly curled and the younger animals are at the centre. A hollow tree might be a suitable habitat for triatomine bugs in that there is an abundant food supply offered by the tamarins. These hollows are high, deep in the trunks and feature a very narrow entrance. The oral route by ingestion of infected triatomine must also be an important route of infection in tamarins, even if triatomine bugs are not very attractive insects because of their scent glands. The simple act of chewing the insect may result in infection, even if the insect is later ejected. Studies on the health of GLTs infected with *T. cruzi* based on electrocardiograms, blood tests and electrophoretograms showed that GLTs appear to support the parasitization by *T. cruzi* without major damage [59].

High rates of *T. cruzi* infection with high parasitemia were also observed in *Sapajus libidinosus* (Cebidae) in the Amazon (Tables 2 and 8). This species is the only New World monkey that uses tools in the natural environment to break nuts. *Sapajus* spp*.* are strictly diurnal, display arboreal habits and spend most of their time foraging and moving through their life area (~300 ha) [60]. They are extremely social mammals that live in groups of up to 50 individuals, led by a larger, older male. They are also cooperative in raising young and finding food. Cebidas are long-living animals and sexual maturity occurs earlier in females (4–5 years) than in males (8–10 years). These primates do not constitute stable pairs and males and females mate with multiple individuals, making determination of paternity difficult [61, 62].

Cebids are difficult subjects for research and we did not have chance to conduct a long-term study. Concerning *T. cruzi* infection, *S. libidinosus* presented TcI in single and mixed infections with *T. rangeli.* Single infections with *T. rangeli* have been also observed (Table 2).

All species of *Sapajus* are omnivorous, but their main diet consists of fruits and insects, in addition to small vertebrates including mammals, which means that the oral route must be important route of infection with *T. cruzi* in this primate group. *Sapajus* spp*.* are arboreal primates and trees are selected in part for food availability. They frequently use palm trees as places to sleep which also provide shelter to triatomine bugs; consequently, infection *via* the contaminative route during the night is also possible.

## Chiroptera

**Fig. 1:**
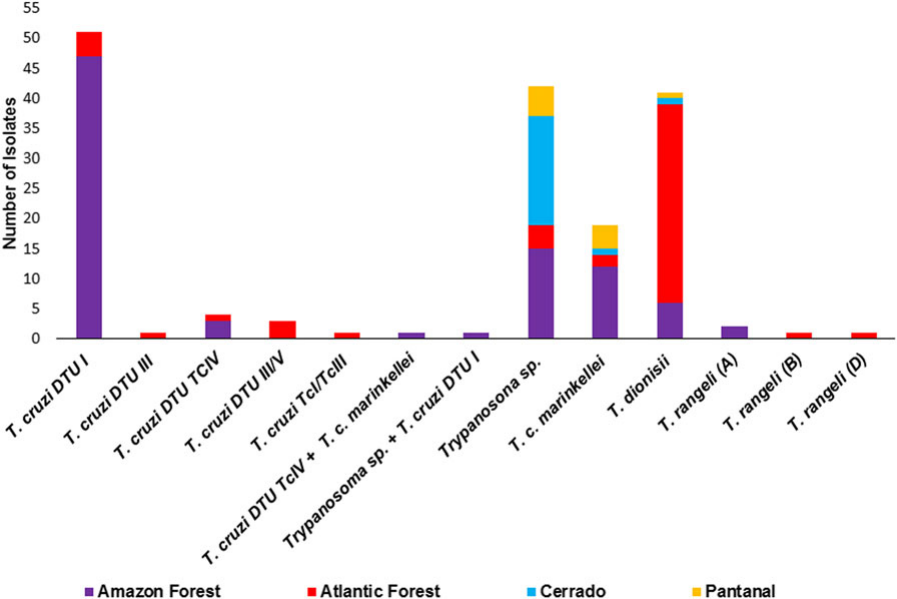
Distribution of *Trypanosoma* species and *T. cruzi* DTUs infecting free-living bats from four Brazilian biomes based on molecular characterization of parasite populations isolated in blood cultures

Table 9 and Fig. 1 show the great diversity of *Trypanosoma* spp. infecting bats and that *T. cruzi* was the most abundant species of *Trypanosoma* (37%). Moreover, the richness of *Trypanosoma* spp. infecting this mammalian taxon is much higher than observed in any other mammalian host. The majority of positive hemocultures of bats revealed the infection by other *Trypanosoma* spp. and not *T. cruzi*. These findings show that bats’ role as *T. cruzi* reservoirs was probably often overestimated at a time when there was still no easy access to sequencing tools. The use of even more sensitive and high-performance tools like NGS would certainly demonstrate even greater diversity of genotypes and species in single and mixed infections in bats. In addition to bats, marsupials have also been found infected with numerous species of *Trypanosoma* including *T. dionisii*, a species considered to be an exclusive parasite of bats until recently [44]. An important question concerns the result of such mixed infections in hosts: what are the consequences in the outcome in such mixed infections?

Bats may be considered as *Trypanosoma* spp. bio-accumulator hosts. There are plenty of recent descriptions of new species and still undescribed taxonomic units of *Trypanosoma* spp. infecting chiropterans all over the world. Chiropterans’ longevity and ability to fly, as well as their capacity in maintaining *T. cruzi* DTUs and other representatives of the *T. cruzi* clade, imply in that these mammals may act as important dispersers of these flagellates. Molyneux [63], and more recently Hamilton et al. [64] proposed bats as the ancestral hosts of the *T. cruzi* clade and that terrestrial mammals became infected with *T. cruzi* as a consequence of several spillover events of the bats trypanosomes. They termed this evolutive path as “the bat seeding hypothesis”. This hypothesis is currently universally accepted based on the description of trypanosomes of the *T. cruzi* clade infecting African bat species in contrast to the low diversity of *T*. *cruzi* clade species other than *T. cruzi* infecting the American terrestrial mammals.

During the last two decades, we examined a total of 1219 bat specimens distributed in 76 genera and 94 species of five Brazilian biomes (Table 9). High infectivity potential for *Trypanosoma* spp., as demonstrated by positive hemocultures, was observed in 14% of these mammals. From these, only 5% were infected with *T. cruzi*, in single or mixed infections. The remaining 9% were infected with other *Trypanosoma* species such as *T. c. marinkellei*, *T. dionisii* and *T. rangeli* (Fig. 1).

The highest rates of *Trypanosoma* spp. infections in bats were observed in the Amazon region (22.6% of positive hemocultures), followed by the Cerrado biome (Table 9). As expected, a higher diversity of bats and *Trypanosoma* spp. species were observed in pristine forest areas in comparison to already altered forest patches. In this sense, a higher variety of bat species was observed in the Amazon in comparison to the Atlantic Forest. This issue is especially striking considering that in the Atlantic Forest the number of examined individuals was almost double that of Amazon (Table 9). Among the infected bats, 62 specimens displayed single or mixed *T. cruzi* DTU infections and the majority of the chiropterans were infected with TcI, but other *T. cruzi* DTUs also may infect bats, demonstrating that these animals are rather resilient to *T. cruzi* genotypes. We did not find *T. cruzi* TcII infecting Chiroptera, and these data raise doubts on what was previously known about bat *Trypanosoma* spp. infections and forces us to rethink the subject.

Of the bat species, members of the genera *Artibeus*, *Carollia* and *Phillostomus* (Phyllostomidae) exhibited the highest rates of infection with the different *Trypanosoma* species. These three genera include generalist species that feed on fruits, but also pollen and insects. Bats are important seed dispersers and probably become infected with *T. cruzi* through the contaminative and oral routes, the latter by ingestion of infected triatomine bugs and mice, as already demonstrated experimentally [65].

Next generation sequencing (NGS) demonstrated to be a promising tool to answer questions concerning *Trypanosoma* specificity, ecology and phylogeny. The possibility of detecting trypanosomatids that are not cultivable in axenic media, as well as the possibility of bypassing the selective forces inherent to isolation methods, are highly informative. Dario et al. [66] conducted a study by metabarcoding DNA samples extracted from blood samples of 34 bats of 13 species captured in Atlantic Forest fragments of Espírito Santo state (southeast region of Brazil). The resulting amplicon sequences could be clustered in 14 operational taxonomic units (OTUs). Five OTUs were identified as *Trypanosoma cruzi* TcI, *T. cruzi* TcIII/V, *T. cruzi marinkellei*, *T. rangeli* and *T. dionisii*, very common trypanosomatid species in Neotropical bats. However, seven clusters demonstrated to be novel genotypes related to the Neotropical bat genotypes. One OTU was identified as a reptile trypanosome and, surprisingly, another OTU was identified as *Bodo saltans*, a free-living Kinetoplastea.

The majority of the bat blood samples demonstrated mixed *Trypanosoma* spp. infections and also mixed OTUs infections by up to eight OTUs. The massive rates of mixed infections are certainly not exceptional in nature and deserve further studies as to evaluate their influence on the course of infection. Actually, these mixed infections cannot be overlooked.

## Conclusions

The set of observations presented here is the most complete and comprehensive study on reservoirs and wild hosts of *Trypanosoma cruzi*. The 20 years of data collection and analyses show that there is no association of *Trypanosoma cruzi* DTU with mammalian host species in their habitat or the biome of their occurrence. In addition, it was possible to conclude that *T. cruzi* is transmitted and remains in the wild even if most of the individuals of the wild mammalian species infected have low parasitemias. Mixed *Trypanosoma cruzi* DTUs and *Trypanosoma* spp. are very common in the wild, and chiropterans and marsupials are the mammals that present the highest diversity of genotypes and infection rates of *T. cruzi* and other species of the genus *Trypanosoma* spp.

## Abbreviations

CI: confidence interval
*df*: degrees of freedom
DTU: Discrete Typing Unit
GLT: Golden Lion Tamarin
HC: Hemoculture
IFAT: Immunofluorescent AssayTests
Labtrip: Laboratório de Biologia de Tripanosomatídeos, IOC/Fiocruz
LIT: liver infusion tryptose
MYBP: million years before present
NGS: next generation sequencing
NNN: Novy-McNeal-Nicolle medium
OTU: Operational Taxonomic Unit
RFLP: restriction fragment length polymorphism
SOP: standard operating procedures
